# Validation of miRNAs as Breast Cancer Biomarkers with a Machine Learning Approach

**DOI:** 10.3390/cancers11030431

**Published:** 2019-03-26

**Authors:** Oneeb Rehman, Hanqi Zhuang, Ali Muhamed Ali, Ali Ibrahim, Zhongwei Li

**Affiliations:** 1 College of Engineering and Computer Science, Florida Atlantic University, Boca Raton, FL 33431, USA; orehman@fau.edu (O.R.); amuhamedali2014@fau.edu (A.M.A.); aibrahim2014@fau.edu (A.I.); 2 Charles E. Schmidt College of Medicine, Florida Atlantic University, Boca Raton, FL 33431, USA; zli@health.fau.edu

**Keywords:** miRNAs, cancer biomarkers, breast cancer detection, machine learning, feature selection, classification

## Abstract

Certain small noncoding microRNAs (miRNAs) are differentially expressed in normal tissues and cancers, which makes them great candidates for biomarkers for cancer. Previously, a selected subset of miRNAs has been experimentally verified to be linked to breast cancer. In this paper, we validated the importance of these miRNAs using a machine learning approach on miRNA expression data. We performed feature selection, using Information Gain (IG), Chi-Squared (CHI2) and Least Absolute Shrinkage and Selection Operation (LASSO), on the set of these relevant miRNAs to rank them by importance. We then performed cancer classification using these miRNAs as features using Random Forest (RF) and Support Vector Machine (SVM) classifiers. Our results demonstrated that the miRNAs ranked higher by our analysis had higher classifier performance. Performance becomes lower as the rank of the miRNA decreases, confirming that these miRNAs had different degrees of importance as biomarkers. Furthermore, we discovered that using a minimum of three miRNAs as biomarkers for breast cancers can be as effective as using the entire set of 1800 miRNAs. This work suggests that machine learning is a useful tool for functional studies of miRNAs for cancer detection and diagnosis.

## 1. Introduction

MicroRNAs (miRNAs) are small non-coding RNA molecules involved in the regulation of gene expression by partially base-pairing to complementary sequences of target messenger RNAs (mRNA), which leads to cleavage and eventual degradation of the target mRNA or translational repression [1]. The objective of this research is to investigate the potential of using a machine learning approach to validate clinically chosen relevant miRNAs as reliable biomarkers for cancer detection and diagnosis.

Calin et al. [2] were among the first who established the relationship between miRNAs and cancers after discovering that mir15 and mir16 are deleted or down-regulated in a majority of chronic lymphocytic leukemia cases. McManus [3] reviewed examples that link miRNA expression to the development of cancer, and proposed a general role of miRNAs in oncogenesis. Further studies conducted on a variety of cancer types reinforced the causal relationship between miRNA and cancer by demonstrating significantly altered expression profiles of miRNAs in cancer as compared to normal tissue [4,5,6]. It was shown that alteration of only a single miRNA can influence cell identity [7]. These observations led to the increasing interests to test the effectiveness of using miRNAs as biomarkers for diagnosing cancer. However, it has been difficult to identify miRNAs that are clearly important for cancer detection as some miRNAs are up-regulated in certain cancers and function as oncogenes while they are down-regulated in others, acting as tumor suppressors. Furthermore, some miRNAs play pivotal roles in cancer development whereas others are less important. This means that identifying relevant miRNAs will be context-sensitive and dependent on the location and type of cancer that is being considered [7,8]. Therefore, computational analysis of large datasets of miRNA and cancer may greatly improve identification of miRNA biomarkers.

Computational methods, especially machine learning, have been applied for cancer detection and diagnosis using miRNAs as biomarkers. Lu et al. [5] used hierarchical clustering on 73 bone marrow samples and determined that miRNA expression distinguishes tumors of different subtypes within acute lymphoblastic leukemia. They constructed a k-NN classifier using lung cancer samples as well as adjacent healthy samples from mice, which achieved a classification accuracy of 100%. Rosenfeld et al. [9] constructed a miRNA-based tissue classifier to identify the source location of metastatic tumors. By using k-NN and decision trees to classify tumors into 22 different tumor origins (classes), they obtained an accuracy of 89% on the validation set. Prior work was also done by Kotlarchyk et al. [10] on the subject of using feature selection on liver, breast and brain cancer datasets. However, the previous studies were limited by the amount of miRNA data that was readily available and the number of miRNAs that were known at the time. With the emergence of the Genomic Data Commons (GDC) Data Portal provided by the National Cancer Institute, the amount of miRNA expression data increased dramatically. With 34 different types of cancers available, more precise experiments could be performed. Waspada et al. [11] used 22 different miRNA expression datasets from the GDC Data Portal and achieved multiple objectives: (i) a multiclass classification combination of all 22 cancer types, (ii) binary classification using only breast cancer data, (iii) binary classification using breast cancer data with the addition of a feature selection step, and (iv) binary classification with miRNAs selected according to clinical research. Cheerla et al. [12] constructed a SVM-RBF classifier trained with various miRNA expression data across 21 different cancer types, achieving an accuracy of 97.2%. They also used feature selection methods to reduce the number of miRNAs to 60 and still achieved a 95.5% overall classification accuracy. Ali et al. [13] used Neighbourhood Component Analysis to extract relevant miRNA features in order to perform subtype classification, achieving around 95% accuracy. These studies demonstrated the potential of improved cancer detection and diagnosis using miRNAs as biomarkers.

This research aims at using a variety of feature selection techniques to select a subset of miRNAs to identify important miRNA features that are crucial in the diagnosis of breast cancer, a cancer that has accumulated ample amount of miRNA data [14]. The miRBase release 22 (version 22) recorded 1917 confirmed mature human miRNAs [15]. For all practical purposes, it is important to narrow down this large number of miRNAs to find the most discriminative subset of miRNA features for the specific tasks we are working on. Since these miRNAs correspond directly as features, we can employ feature selection methods to remove irrelevant and redundant ones. In machine learning, feature selection is a method of selecting a subset from a given feature set based on a certain set of criteria without transformation of the original features, which preserves the interpretation of the results. This prevents overfitting and improves classification performance especially with gene expression data, which usually has a large number of features. In order to determine the best subset of features, we focus only those miRNAs which have been verified clinically. This allows us to focus on miRNAs that have been deemed relevant in the laboratory. We then apply feature selection methods on this reduced subset of miRNAs to determine which are more important for cancer detection. Even though quite a few miRNAs have been clinically shown to be linked to breast cancer, we aim to show with a machine learning approach that not all miRNAs are equally important as a cancer biomarker, even among those clinically selected ones.

## 2. Methodology

**Figure 1:**
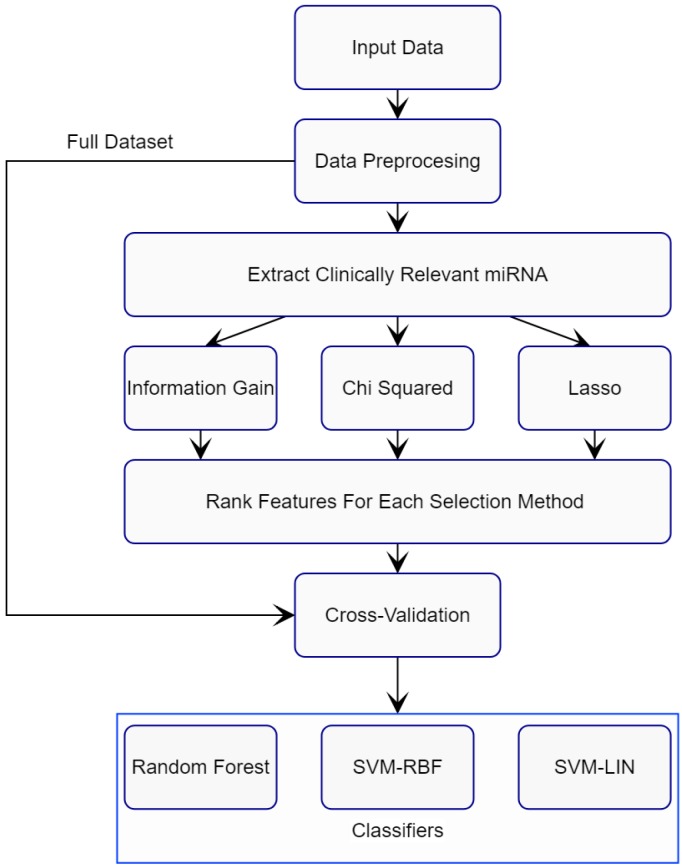
Schematics for Cancer Detection with Machine Learning.

Our approach for validating clinically selected miRNAs for cancer detection and diagnosis is summarized in Figure 1.

The algorithm is divided into two stages: training and verification. In the training stage, the first step is to clean up the miRNA row data by removing rows with all zero values. We then keep only those miRNAs that have been identified via wet lab as possible biomarkers for breast cancer detection. These biomarkers are classified as clinically verified miRNAs. The list of clinically verified miRNAs identified in the literature is shown in Table 1. Three feature selection methods, Information Gain, Chi Squared, and LASSO, are applied to independently rank the importance of miRNAs. The resulting feature vectors are then fed to classification algorithms. For this purpose, two classifiers, Random Forest and Support Vector Machines (SVMs), are applied to train the model. Feature selection is performed on the selected subset of the breast-cancer dataset and these miRNA features are ranked by each feature selection method individually. From these ranked features, different subsets were selected and were then fed into the classification algorithms. The performance of the miRNAs is then evaluated based on certain performance metrics which will be introduced later.

In the next subsections, we discuss the techniques used for both feature selection and classification.

### 2.1. Feature Selection

As has been mentioned before, the objective of feature selection is to identify the specific miRNAs that are most effective in discriminating normal and cancerous tissues. Since the dimensionality of expression data is large in relation to the number of samples, it is easy for classifiers to over-fit, therefore a reduction in feature size will alleviate that problem. Another important aspect of feature selection versus using certain dimensionality reduction techniques, such as Principal Component Analysis, Discrete Cosine Transform and Wavelet Transform, is that we can preserve the original features as opposed to mapping them to a different representation.

In this section, three popular feature selection techniques, Information Gain, Chi-Squared Feature Selection, and Least Absolute Shrinkage and Selection Operator, are reviewed. For a detailed discussion of these methods, readers are referred to [16,17,18].

#### 2.1.1. Information Gain

Information Gain (IG) is a feature selection method based on Information Theory, which measures the reduction of entropy that occurs by having knowledge of a feature, *A*. For a dataset *X* with n class labels, the Shannon entropy, which is a measure of unpredictability, is given by the following equation,

(1) $$H\left(X\right)=-\sum_{i=1}^{n}p_{i}\log_{2}p_{i}.$$

where $p_{i}$ is the probability of class *i* in the data set *X*. IG is the reduction of entropy that is achieved by knowing the feature *A*, shown by the following equation,

(2) IG(X,A)=H(x)−H(X|A).

where,

(3) $$H\left(X|A\right)=\sum_{i=i}^{v}\frac{X_{i}}{X}H\left(X_{i}\right).$$

where $X_{i}$ is a subset of *X* containing a distinct value of *A*, *v* is the number of distinct values present in *A* and $H(X_{i})$ is the entropy of the *i*-th subset created by splitting *X* on feature *A*. Therefore, IG can be seen as the difference between the prior entropy and the entropy after splitting the original dataset based on the feature *A*.

#### 2.1.2. Chi-Squared Feature Selection

Chi-squared (CHI2) is another feature selection method which evaluates features with respect to the classes. It is a statistical test to determine the dependency of a feature on the class label. We can discard features that do not show dependency and extract the relevant features that are useful for classification. The range of continuous valued features needs to be discretized into intervals.

(4) $$\chi^{2}=\sum_{i=1}^{C}\sum_{j=1}^{I}\frac{{\left(A_{ij}-E_{ij}\right)}^{2}}{E_{ij}}$$

where *C* is the number of classes, *I* is the number of intervals, $E_{ij}$ is the expected number of samples, $A_{ij}$ is the number of samples of the $C_{i}$ class within the *j*-th interval. The larger the value of $\chi^{2}$, the more information the corresponding feature provides.

#### 2.1.3. Least Absolute Shrinkage and Selection Operator

Least Absolute Shrinkage and Selection Operator (LASSO) is a regularization and variable selection method for statistical models. LASSO minimizes the sum of squared errors while also being subject to a constraint on the sum of the absolute values of the regression coefficients, which is described by,

(5) $$\min_{\beta_{0},\beta }\sum_{i=1}^{N}{\left(y_{i}-\beta_{0}-x_{i}^{T}\beta \right)}^{2}s.t.\sum_{j=1}^{p}\left|\beta_{j}\right|\leq t.$$

where *N* is the number of cases, *p* is the number of features, ($\beta ,\beta_{0},\beta_{j}$) are regression coefficients, $y_{i}$ is the *i*-th predicted output and $x_{i}$ is the *i*-th set of features. By tuning the parameter *t*, we can choose the best performing features, as the less predictive coefficients will go to zero.

### 2.2. Classification

After feature selection, one can apply classification algorithms to determine if the target is cancerous or not. In this section, two classifiers, Random Forest and Support Vector Machine (SVM), are overviewed [11,18].

#### 2.2.1. Random Forest

The Random Forest (RF) algorithm is an ensemble classifier that generates multiple decision trees, including weak classifiers learned on a random sample from the data. The classification of a new sample is done by majority voting of the decision trees. Random Forest is constructed in the following manner. Assume that the given training set has *N* cases, each with *M* features. Each decision tree is grown as follows: First, *n* samples are selected at random with replacement from the training set. At each node of the tree, m<<M of the features are selected at random. The best split on these *m* features, based on some objective function (for instance, Information Gain), is used to perform a binary split on that node. This process is repeated until a predefined minimum node size is reached. Classification of new data is done through majority votes by aggregating the predictions of all the decision trees.

#### 2.2.2. Support Vector Machine

Support Vector Machine (SVM), a supervised machine learning method, aims to design an optimum hyperplane that separates the input features into two difference classes for binary classification. The best solution maximizes the margin, defined by so-called support vectors, between both classes. Given the miRNA data consists of n feature vectors, ($x_{i},y_{i}$), where $y_{i}\in \left\{+1,-1\right\}$, one can construct an optimization problem in which the distance between the margins is maximized by minimizing the following equation,

(6) $$\frac{1}{2}{\left||w|\right|}^{2}$$

under the following constraint,

(7) $$y_{i}\left(wx_{i}+b\right)-1\geq 0.$$

where *w* is the weights vector which dictates the margin size and *b* is the bias, which shifts the hyperplane boundary. In the case of non-linearly separable data, kernel functions can be used to map the input space to a higher dimensional feature space to allow for a linear separation. A popular kernel function, the Radial Basis Function (RBF), is given as follows:

(8) $$k\left(x_{i},y_{i}\right)=\exp\left(-\gamma ||x_{i}-x_{j}{\left|\right|}^{2}\right)$$

where γ is a hyperparameter that controls the error due to bias and variance. We will use both linear SVM as well as SVM with a radial basis function kernel (RBF) in our experimentation.

## 3. Results

The microRNA expression dataset for breast cancer was obtained from the National Cancer Institute’s Genomic Data Commons Data Portal [19]. This dataset consists of 1207 patient samples with 1881 miRNA features, containing 1103 primary solid tumor samples, 7 metastatic samples and 104 healthy samples. The dataset is imbalanced as there are many more number of cancerous samples compared to healthy samples.

The dataset included raw read counts as well as counts normalized to reads per million mapped reads (RPM). The metastatic samples were combined with the solid tumor samples as one class, since metastatic cancer tissue retained most of the genomic features of the source tumor [20]. The log2 of the RPM values was taken plus a pseudo count of 1 and then the values were standardized to zero mean and unit variance. Zero values were also removed which further reduced the number of miRNA features to 1626. Then miRNAs that were not identified by Table 1 were removed.

The dataset was run through the classifiers first without feature selection and then with feature selection using different feature selection methods (IG, LASSO and CHI2, see Section 2.1). In the experiment, we grouped miRNAs into subsets of 3, 5, and 10 members and test their effectiveness in identifying cancer using different feature selection procedures with different classifiers. Also, 10-fold validation was performed throughout. The 10-fold validation is a technique in which in each trial, 90% of the data samples are used for training and the remaining 10% for testing; the process is repeated 10 times, ensuring that all samples are tested once. We chose to only utilize cross-validation and neglect the other steps outlined by the Data Analysis Protocol (DAP), which is outlined by the US-FDA MAQC-II initiative [21]. This is because the focus of this paper is to demonstrate that a small subset of miRNAs can be used to detect cancer with 10-fold validation [22].

Due to the nature of the unbalanced dataset, using only accuracy as a performance metric may misrepresent the performance of our classifiers. In the experiment, we establish the outcomes using the following measures: True positive (TP), False Positive (FP), True negative (TN) and False negative (FN). Here the positive class means a tumorous sample and the negative class is non-cancerous (healthy). Specificity is defined as,

(9) $$\frac{TN}{TN+FP}$$

which is the proportion of non-cancerous samples correctly identified. Sensitivity is,

(10) $$\frac{TP}{TP+FN}$$

which tests the ability for the cancerous samples to be correctly identified. In addition, Accuracy is simply,

(11) $$\frac{TP+TN}{TP+TN+FP+FN}$$

which tests the overall ability to different between healthy and cancerous samples. We also calculate Area Under Curve (AUC).

Table 2 shows the performance of different feature selection techniques vs different classifiers. In the table, the first column indicates which classifier was used in the experiment. The second one lists the feature selection method along with the number of miRNAs in each group. For instance, IG-10 means that Information Gain is used for feature selection, and miRNAs are grouped into 10 each. Other columns show the performance of the feature selection algorithm teamed up with the classifier.

Examining Table 2, we can see that the performance metric that fluctuates the most is the specificity. Since it is possible to achieve a high accuracy even while misclassifying all of the minority class, we need to look at a performance metric that can give us a more meaningful result. Note that the classification accuracy across all selections is practically the same, which confirms its ineffectiveness as a performance metric. The difficulty of this dataset lies in correctly classifying its minority class. We can see a trend of improved sensitivity values when feature selection is used for the RF and SVM-RBF classifiers. This reinforces the notion that there are redundant and irrelevant features present in the dataset and that we may be able to achieve better results with a handful of features rather than the original 1881. We also observed a marked improvement in terms of Specificity by applying any type of feature selection (across all subsets).

Examining the results from Table 2, one can also see that even using a small fraction of the entire feature set, one may obtain very good classification results. This means that clinically one may only need to focus on just a few miRNAs to diagnose a patient. In the next section, we ranked the importance of individual miRNAs under different feature selection techniques. Table 3 show the test results. In this table, miRNAs are ranked in a top-down order under different feature selection algorithms. This means that the miRNAs listed in the top of the table provide better detection performance.

We can see that the subsets of Info Gain and CHI2 are remarkably identical, while overlap six features Lasso, as shown in Table 3.

We can also use feature selection to rank those clinically-selected miRNAs. After ranking, we can verify our results by taking different subsets and testing their performance for cancer detection. We begin by choosing the top four miRNA features in subsets of IG and CHI2, ranked 1–3 as Subset #1. We then slide down by choosing ranked 2–4 miRNAs as Subset #2, and so on. In this way, we obtain eight different subsets, shown in Table 4. We choose four miRNAs as our threshold to mirror our previous experiment as that served as a good limit before performance degradation.

**Figure 2:**
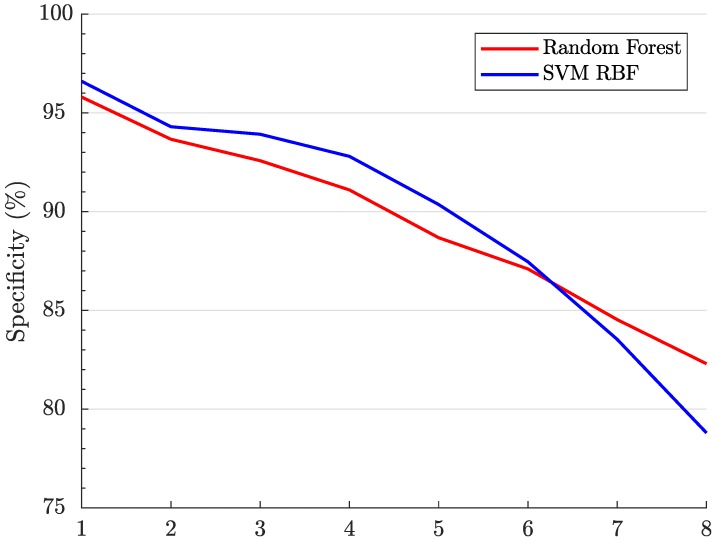
Specificity Across Different Clinical miRNA Subsets.

We can now evaluate these data sets with both RF and SVM algorithms. The Specificity has been plotted across the eight subsets, shown in Figure 2.

Interestingly, we observe a downward trend as the subset index (the horizontal axis) goes up, which demonstrates a decrease in classifier performance as we go from Subset #1 to #7. The results strongly suggest that miRNAs that are ranked higher are better biomarkers for breast cancer detection than the ones on the bottom in the list.

## 4. Conclusions

Our results in this work validate clinically-chosen miRNAs as biomarkers for breast cancer detection with a machine learning approach. It demonstrates that by ranking miRNAs using feature selection methods, one is able to determine the best performing miRNAs for breast cancer detection among those clinically verified ones. Our tests have also demonstrated that with merely three selected miRNAs as biomarkers, the classifiers can still produce nearly optimal results in breast cancer detection, in comparison to the use of many more miRNAs.

There are multiple avenues to pursue regarding further work. Specifically, one may extend the framework to identify discriminative miRNAs that indicate different stages of cancer progression if features are available in the datasets. One can also extend these ideas for other cancer types. These other datasets may have common characteristics which can be leveraged using machine learning techniques. With machine learning, one may be able to overcome the problems caused by very small number of samples in cancer datasets. More importantly, the ability of machine learning to classify breast cancer related miRNAs demonstrated here may lead to future development of robotic methods for de novo identification of miRNA biomarkers for other diseases with or without laboratory data.

## Figures and Tables

**Table 1:** Clinically Verified miRNA.

miRNA [14]			
hsa-mir-10b	hsa-let-7d	hsa-mir-206	hsa-mir-34a
hsa-mir-125b-1	hsa-let-7f-1	hsa-mir-17	hsa-mir-27b
hsa-mir-145	hsa-let-7f-2	hsa-mir-335	hsa-mir-126
hsa-mir-21	hsa-mir-206	hsa-mir-373	hsa-mir-101-1
hsa-mir-125a	hsa-mir-30a	hsa-mir-520c	hsa-mir-101-2
hsa-mir-17	hsa-mir-30b	hsa-mir-27a	hsa-mir-146a
hsa-mir-125b-2	hsa-mir-203a	hsa-mir-221	hsa-mir-146b
hsa-let-7a-2	hsa-mir-203b	hsa-mir-222	hsa-mir-205
hsa-let-7a-3	has-mir-213	hsa-mir-200c	
hsa-let-7c	hsa-mir-155	hsa-mir-31	

**Table 2:** Performance Metrics across different thresholds of miRNA Features (3, 5, 10).

Classifier	Method	Accuracy	Sensitivity	Specificity	AUC
RF		0.996	1.000	0.952	0.999
	IG-10	0.995	0.998	0.962	0.996
	IG-5	0.996	0.997	0.977	0.998
	IG-3	0.997	0.997	0.990	0.999
	CHI2-10	0.995	0.999	0.952	0.995
	CHI2-5	0.996	0.999	0.979	0.996
	CHI2-3	0.996	0.997	0.981	0.999
	LASS-10	0.996	0.998	0.971	0.997
	LASS-5	0.995	0.997	0.965	0.998
	LASS-3	0.994	0.997	0.962	0.999
SVM-RBF		0.989	1.000	0.875	0.938
	IG-10	0.994	0.998	0.952	0.995
	IG-5	0.996	1.000	0.990	0.985
	IG-3	0.998	0.998	0.990	0.980
	CHI2-10	0.994	0.999	0.951	0.995
	CHI2-5	0.996	0.998	0.983	0.993
	CHI2-3	0.998	0.999	0.990	0.980
	LASS-10	0.995	0.998	0.962	0.996
	LASS-5	0.995	0.999	0.974	0.985
	LASS-3	0.996	0.999	0.962	0.980
SVM		0.997	0.999	0.971	0.985
	IG-10	0.997	0.999	0.971	0.997
	IG-5	0.997	0.999	0.985	0.989
	IG-3	0.998	0.999	0.990	0.981
	CHI2-10	0.997	0.999	0.971	0.997
	CHI2-5	0.996	1.000	0.988	0.987
	CHI2-3	0.998	0.999	0.990	0.991
	LASS-10	0.994	0.997	0.962	0.996
	LASS-5	0.995	0.999	0.956	0.993
	LASS-3	0.997	1.000	0.962	0.981

**Table 3:** Top Ranked Features Under Different Feature Selection Techniques.

Info Gain	CHI2	Lasso
hsa-mir-10b	hsa-mir-10b	hsa-let-7a-3
hsa-let-7c	hsa-let-7c	hsa-let-7c
hsa-mir-145	hsa-mir-145	hsa-let-7d
hsa-mir-125b-1	hsa-mir-125b-2	hsa-mir-101-1
hsa-mir-125b-2	hsa-mir-125b-1	hsa-mir-10b
hsa-mir-335	hsa-mir-335	hsa-mir-125b-2
hsa-mir-126	hsa-mir-126	hsa-mir-145
hsa-mir-125a	hsa-mir-125a	hsa-mir-206
hsa-let-7a-2	hsa-let-7a-2	hsa-mir-27b
hsa-let-7a-3	hsa-let-7a-3	hsa-mir-335

**Table 4:** Subset Selection of Ranked miRNA.

Subset 1	Subset 2	Subset 3	Subset 4	Subset 5	Subset 6	Subset 7	Subset 8
hsa-mir-10b	hsa-let-7c	hsa-mir-145	hsa-mir-125b-1	hsa-mir-125b-2	hsa-mir-335	hsa-mir-126	hsa-mir-125a
hsa-let-7c	hsa-mir-145	hsa-mir-125b-1	hsa-mir-125b-2	hsa-mir-335	hsa-mir-126	hsa-mir-125a	hsa-let-7a-2
hsa-mir-145	hsa-mir-125b-1	hsa-mir-125b-2	hsa-mir-335	hsa-mir-126	hsa-mir-125a	hsa-let-7a-2	hsa-let-7a-3

## References

[1] He L., Hannon G.J. (2004). MicroRNAs: Small RNAs with a big role in gene regulation. Nat. Rev. Genet..

[2] Calin G.A., Dumitru C.D., Shimizu M., Bichi R., Zupo S., Noch E., Aldler H., Rattan S., Keating M., Rai K. (2002). Frequent deletions and down-regulation of micro- RNA genes miR15 and miR16 at 13q14 in chronic lymphocytic leukemia. Proc. Natl. Acad. Sci. USA.

[3] McManus M.T. (2003). MicroRNAs and cancer. Semin. Cancer Biol..

[4] Croce C.M. (2009). Causes and consequences of microRNA dysregulation in cancer. Nat. Rev. Genet..

[5] Lu J., Getz G., Miska E.A., Alvarez-Saavedra E., Lamb J., Peck D., Sweet-Cordero A., Ebert B.L., Mak R.H., Ferrando A.A. (2005). MicroRNA expression profiles classify human cancers. Nature.

[6] Volinia S., Calin G.A., Liu C.G., Ambs S., Cimmino A., Petrocca F., Visone R., Iorio M., Roldo C., Ferracin M. (2006). A microRNA expression signature of human solid tumors defines cancer gene targets. Proc. Natl. Acad. Sci. USA.

[7] Jansson M.D., Lund A.H. (2012). MicroRNA and cancer. Mol. Oncol..

[8] van Schooneveld E., Wildiers H., Vergote I., Vermeulen P.B., Dirix L.Y., Van Laere S.J. (2015). Dysregulation of microRNAs in breast cancer and their potential role as prognostic and predictive biomarkers in patient management. Breast Cancer Res..

[9] Rosenfeld N., Aharonov R., Meiri E., Rosenwald S., Spector Y., Zepeniuk M., Benjamin H., Shabes N., Tabak S., Levy A. (2008). MicroRNAs accurately identify cancer tissue origin. Nat. Biotechnol..

[10] Kotlarchyk A., Khoshgoftaar T., Pavlovic M., Zhuang H., Pandya A. (2011). Identification of microRna biomarkers for cancer by combining multiple featureselection techniques. J. Comput. Methods Sci. Eng..

[11] Waspada I., Wibowo A., Meraz N. (2017). Supervised Machine Learing Model for microRNA Expression Data in Cancer. Jurnal Ilmu Komputer dan Informasi.

[12] Cheerla N., Gevaert O. (2017). MicroRNA based Pan-Cancer Diagnosis and Treatment Recommendation. BMC Bioinf..

[13] Muhamed Ali A., Zhuang H., Ibrahim A., Rehman O., Huang M., Wu A. (2018). A Machine Learning Approach for the Classification of Kidney Cancer Subtypes Using miRNA Genome Data. Appl. Sci..

[14] Fu S.W., Chen L., Man Y.G. (2011). miRNA Biomarkers in Breast Cancer Detection and Management. J. Cancer.

[15] miRBase: The microRNA Database. http://www.mirbase.org/.

[16] Saeys Y., Inza I., Larranaga P. (2007). A review of feature selection techniques in bioinformatics. Bioinformatics.

[17] Ghosh D., Chinnaiyan A.M. (2005). Classification and selection of biomarkers in genomic data using LASSO. J. Biomed. Biotechnol..

[18] Razak E., Yusof F., Raus R.A. Classification of miRNA Expression Data Using Random Forests for Cancer Diagnosis. Proceedings of the 2016 International Conference on Computer and Communication Engineering (ICCCE).

[19] The Cancer Genome Atlas. http://cancergenome.nih.gov/.

[20] Liu G., Zhan X., Dong C., Liu L. (2017). Genomics alterations of metastatic and primary tissues across 15 cancer types. Sci. Rep..

[21] Shi L., Campbell G., Jones W.D., Campagne F., Wen Z., Walker S.J., Su Z., Chu T.M., Goodsaid F.M., Pusztai L. (2010). The MicroArray Quality Control (MAQC)-II study of common practices for the development and validation of microarray-based predictive models. Nat. Biotechnol..

[22] Cruz J.A., Wishart D.S. (2006). Applications of Machine Learning in Cancer Prediction and Prognosis. Cancer Inf..

